# Texas State Medical Association—Twenty-sixth Annual Meeting

**Published:** 1894-05

**Authors:** 


### Society Notes.

### TEXAS STATE MEDICAL ASSOCIATION—TWENTY-SIXTH ANNUAL MEETING.

### Synopsis of Proceedings.

Pursuant to call, the Texas State Medical Association held its 26th annual meeting in the Senate Chamber, at the Capital City, April 24, 25, 26 and 27, ult. The attendance was smaller than for years,—attributable to "financial stringency,"—about one hundred members and delegates being present the first day; increased next day to perhaps one hundred and fifty. As usual, there were about fifty old stand-bys, who attend all meetings; but it was noticeable that there was a large attendance of doctors

NOTE.—The JOURNAL was promised a copy of the minutes of the meeting, and the forms were held back till the 8th instant, waiting in vain for copy. Could delay no longer, and had to prepare the above report from imperfect and disconnected notes of our own, and from the worse than imperfect reports in the Statesman. We have omitted much of interest, from force of circumstances; and if any gentleman's paper has not been mentioned, we hope it will be understood that it was unintentional.—ED.

whose faces were not familiar—new doctors and young doctors. The Association made some valuable acquisitions to its membership.

For the first time within the memory of this scribe, the Convention was opened without prayer and asking Divine aid. Rev. G. W. Briggs, the popular and eloquent Methodist minister of Austin, had been invited to open the session, and the committee relied so fully upon him that an alternate or substitute was not provided, and he disappointed us.

Chairman of Arrangement Committee Denton called the meeting to order, and introduced Hon. John McDonald, the ugly but popular Mayor of Austin, who tendered visitors an invitation to take all they could get, and make themselves at home. After him came Judge Henderson, who welcomed the doctors in a speech which seemed to strike the hearers about right; then the Convention proceeded to business.

### SECRETARY'S ANNUAL REPORT.

The annual report of the Secretary shows the following result:

| | |
|---|---|
| Members on the roll at last report | 416 |
| Honorary members on the roll at last report | 21 |
| Total | 437 |
| On the roll May 1, 1893: | |
| Members | 406 |
| Members added (name dropped by mistake) | 2 |
| Members, honorary | 23 |
| Total | 431 |
| New members elected at Galveston | 37 |
| Honorary | 2 |
| Total | 470 |
| Dropped for non payment of dues: | |
| Resigned, Dr. J. V. Spring, of San Antonio | 1 |
| Resigned, Dr. J. L. Cunningham, of Ft. Worth | 1 |
| Resigned, Dr. J. M. Litten, of Austin | 1 |
| Total | 3 |

Lost members by death, Dr. C. F. Paine, of Comanche, who died September 13, 1893.

These ex-members have died: Dr. M. H. Oliver, of Ennis, died September 5, 1893; Dr. Jno. L. Wagley, Cleburne, December 13, 1893; Dr. H. W. Waters, of Independence, January 4, 1893. The Secretary adds that there may be omissions in this list, as he is dependent for data upon the files of the medical journals, as no one interested ever takes the trouble to furnish him with direct information.

The Transactions for 1893 are as follows: Soon after adjournment last year, bids for printing the Transactions were submitted from the following firms: Robt. Clarke & Co., Clarke & Courts, Strickland Printing Co., and Knoff Bros. The latter being the lowest bidders, and they having demonstrated their ability to do the work satisfactorily the previous year, the contract was awarded to them upon a basis of $1.49 per page.

Details for the publisher's bill are as follows:

| | |
|---|---|
| Printing 500 copies, 448 pages at $1.49 per page | $667 52 |
| Five engravings | 42 55 |
| Extra paper, printing and insertion of cuts | 31 60 |
| Ten extra copies | 17 90 |
| Tabular work and extra composition | 24 45 |
| Total | $784 02 |
| Postage | 77 01 |
| Wrapping, addressing and delivering | 20 00 |
| Total | $881 03 |
| Cost per volume, about | $ 1 54 |
| Cost per volume, delivered | 1 80 |
| Number of volumes delivered to members | 431 |
| Number of volumes sold | 6 |
| Number of volumes sent to public libraries | —— |
| Medical journals and others | 55 |
| Number of volumes on hand | 19 |

The increased cost of the Transactions of '93 over that of '92 was simply due to the fact that it was a very much larger book, viz.: 138 pages more. At the outset of publication it became a question whether the Treasurer would have the necessary funds to pay the bill, and it was thought that he would have to levy a small assessment; fortunately, we did not have to do this, but last year's experience forces upon us the question of economy. I would suggest, as one means to this end, that authors in the future be required to pay for their own illustrations. If the volume this year should equal or exceed in cost those of last year, we may have to consider the question of leaving off the cloth binding, by which means about seven per cent. of the total cost might be saved. The questions are presented for your consideration now, as action may be demanded upon them very soon. While it is incumbent for us to maintain a high standard of excellence both in the appearance and quality of our annual volume of transactions, at the same time it is absolutely necessary to keep expenses within the bounds of our receipts.

Excuse me for once more harping upon an old theme, viz., the care requisite in the selection of officers; more especially chairmen of sections. In my opinion, it would be a wise innovation to have the latter elected upon the recommendation of an executive committee, instead of trusting to the hap-hazard action likely to result from the present method.

The programme for this meeting shows a commendable effort upon the part of some chairmen to secure papers, while others have given no evidence of the slightest interest in the matter, not even replying to my letters upon the subject.

Respectfully submitted,

H. A. WEST, M. D., Secretary.

The Secretary's report was referred to a committee: Drs. M. M. Smith, P. C. Coleman and A. M. Alexander. They, later, reported it correct and advised its adoption.

TREASURER'S REPORT.

The report of the Treasurer, Dr. J. Larendon, shows the following statement:

*Receipts for 1893:*

| | |
|---|---|
| May 1, to cash balance on hand as per last annual report.................................... | $ 121 01 |
| April 21, 1894, to cash collected from members for dues up to date.................................... | 1,535 00 |
| Total.................................... | $ 1,656 01 |

*Disbursements:*

| | |
|---|---|
| May 4, 1893— | |
| By cash paid H. A. West, for Secretary's salary...$ | 200 00 |
| By cash paid to Publishing Company............ | 300 00 |
| Py cash paid to J. Larendon, for Treasurer's salary | 150 00 |
| By cash paid to Stenographer.................... | 50 00 |
| By cash paid by Treasurer for postage.... ..... | 10 00 |
| By cash paid H. A. West, for stationery.......... | 5 00 |
| By cash paid by Treasurer for stationery and printing.................................... | 12 00 |
| July 6,— | |
| By cash paid H. A. West, for stationery, etc...... | 13 60 |
| August 30,— | |
| By cash paid Knapp Bros. for printing Transactions | 881 03 |
| By cash paid for registered letter................ | 08 |
| April 21, 1894— | |
| By cash balance on hand........................ | 34 40 |
| Total.................................... | $ 1,656 01 |

Referred to a committee. Adopted upon the report of committee, who found it correct, of course. [The Treasurer has served about twenty-five years as such and there has never been a "t"

not crossed nor an "i" not dotted in his accounts in all these years. May he live to serve twenty-five years more.—ED.]

Next came the

### PRESIDENT'S ADDRESS AND RECOMMENDATIONS.

*To the Members of the State Medical Association:*

COMPANIONS:—It affords me great pleasure to meet and greet so many of my old and tried friends from the different sections of this great State. We have been toiling for a quarter of a century, I hope, with no greater disturbances than are usual to frail humanity. I feel now that we are a harmonious brotherhood, and that we shall meet at this meeting in the beautiful capital of our State, have a joyous time in renewing our pledges of fidelity and brotherly love. We have finished our boyish misunderstandings and "scraps," and arrived at years of full maturity to do the work of men in a most harmonious manner. Let us rejoice that we have reached thus far with a faithful few who have weathered the storms and reached the haven of safety of the first quarter. Our faithful secretary, in his last annual report, gives the number of enrolled members at 417; now, if these were all delegates, as they should be, representing five members of a local or district society, we would have here to-day represented about 2000 of the 4000 or 5000 regular medical men of the State. If this were true it would make a brilliant showing for a quarter of a century's work. It leaves a large field still uncultivated in our territory. The question naturally arises, can we do anything to remedy this state of affairs; ought we not at this meeting adopt some measure to induce them to unite with us in the good work we are trying to do? Would it not be well to send them some pressing invitations to organize and send representatives to this association? Can we not in some way induce them to take more interest in the elevation of the profession and the advancement of science? Who will go as missionaries to them?

Dr. R. M. Swearingen, our efficient health officer, in reply to a letter written him, asking him what legislation was necessary to make our present health department a model, replied:

First—A law by which mortuary and vital statistics can be gathered.

Second—Authority given the State Health Officer to appoint an expert in microscopy and chemistry, in ascertaining the causes of diseases and epidemics, when in his judgment such investigations are necessary.

I would recommend that this Association instruct its committee on legislation to make their best effort to procure such enactment; would also recommend that the same committee be instructed to secure a law creating a board of examiners, before whom anyone professing to practice medicine in this State shall go for examination before being admitted to practice. I am not certain that we ought to ask a separate board for the different schools responsible for its own work. I would likewise recommend that the same committee do their best to get an act passed requiring that every patent nostrum offered for sale in this State shall have on every package a formula of its contents printed in plain English, so that all can understand what they are swallowing. Dr. R. M. Swearingen, in Fort Worth in 1890, so perfectly and beautifully discussed the subject of State laws regulating the practice of medicine that there is nothing left to be said. He refers the whole matter to the people and let them demand it for their protection, not ours. Hence, we urge the organization of our profession that we may influence the people to make the demand. If it be true that the people rule, let us refer all such matter to them. Let us hope that this session, in this beautiful capital city, may be the best and most profitable ever held, and prove a pivotal one of a new and brighter era for all time to come.

### THE JUDICIAL COUNCIL.

Upon the conclusion of President Sears' address, the roll of the Judicial Council was called, and only two members, Drs. Loggins and ———, responded.

The chair then appointed the following substitutes, to act until the regulars arrived:

Doctors R. M. Swearingen, T. J. Bennett, T. D. Wooten, P. C. Coleman, M. D. Knox, E. L. Menafee, R. C. Nettles, J. F. Y. Paine, W. P. Burts and Sam R. Burroughs.

Invitations were received from Dr. White, Superintendent of the Insane Asylum, inviting the physicians to visit that institution, and also from Dr. Wooten, President of the Board of Regents, inviting them to visit the University of Texas during their stay in the city.

Dr. Denton announced that the printed program had been changed to the extent that the excursion up the lake on the steamer Ben Hur, would be given to-morrow, Thursday, instead of Friday afternoon, and that the banquet at the Driskill would be given also Thursday evening instead of to-morrow evening, as previously announced. After this the Convention stood adjourned until 2:30.

---

### AFTERNOON SESSION.

Convention met again in the Senate Chamber at 2:30 o'clock with President Sears in the chair.

Dr. J. C. Jones, of Gonzales, the Chairman of the Section on Practice of Medicine, being absent, the chair appointed Dr. J. W. Carhart as Chairman, and Dr. J. S. Price was appointed Secretary in the place of Dr. I. E. Clark, the regular Secretary, who was also absent.

The first order of business for the afternoon was the reading by Dr. H. A. West, of Galveston, of a paper entitled the

BACTERIOLOGICAL ASPECT OF CROUPOUS PNEUMONIA.

The paper was received, and on motion, was referred to the Publishing Committee.

Dr. Cerna, of Galveston, discussed the paper, endorsing Dr. West's plan to the letter. President Sears also arose for a brief discussion of the paper. He advocated an entirely different plan from the one laid down in Dr. West's paper. Dr. Denton, of Austin, also came to the front in a brief discussion, commending the paper in the main.

Dr. Britton, of Arlington, spoke on Dr. West's paper. The doctor advocated a go between treatment. He said Dr. West was one extreme, and Dr. Sears was the other, and he advocated a consideration of both and a treatment that split the difference.

Dr. Matthews, of Austin, also discussed the paper briefly, advocating the use of alcoholic stimulants in the treatment of croupous pneumonia.

Dr. Q. C. Smith, of Austin, spoke in reference to alcoholic treatment, favoring its use if knowledge of its use goes with it.

Dr. J. W. Carhart, LaGrange, spoke on the paper also, opposing the use of alcoholic stimulants.

Dr. Cerna, of Galveston, again spoke, favoring the paper and answering the adverse arguments offered by the other speakers.

Dr. Alexander discussed the paper briefly, and Dr. West then arose in defense of his paper. He said he had very little to say in addition to the facts laid down in his paper. He had quoted authorities in his paper that knew what they were talking about, and he considered it the height of presumption for gentlemen who knew nothing at all about the matter, to get up and oppose it and say it was wrong just because it did not suit their liking. He added further, that he considered his authorities so sound that it was absolutely unnecessary for him to say more on the subject.

The next paper was that of Dr. James H. Bell, of Philadelphia, who presented a very ably written paper on the subject

THE EMBARRASSMENTS OF MEDICINE.

On motion of Dr. Britton, the paper was referred to the Publishing Committee, and a vote of thanks offered offered Dr. Bell for his paper.

The next paper read was by Dr. David Cerna, of Galveston. Choosing as his subject,

THE THERAPEUTIC USES OF SPARTEINE, he gave a very exhaustive review thereon.

On motion of Dr. Britton, the paper was referred to the Publishing Committee. Dr. Britton also took occasion to compliment Dr. Cerna on his paper.

Dr. D. R. Wallace, of Waco, was next on the program with a paper touching on the subject

HYDROTHERAPY.

The subject was briefly but exhaustively discussed, and was listened to with marked attention by all present.

On motion, the paper was referred to the Publishing Committee. Dr. Britton arose and asked if the Convention knew him (the reporter must explain to those who read this who were not present that Dr. Britton is the funny man of the profession). Being informed that they did, by this time, he proceeded to say that he endorsed Dr. Wallace's paper as far as it referred to the use of water, but condemned him when he proposed to do away with all other treatments. Dr. Britton added further that it was not right; that he was opposed to it, and what he was opposed to was no good and everybody knew it.

Dr. Cerna questioned the practicability of using water to the exclusion of all else.

Dr. West endorsed Dr. Wallace's paper in a brief talk, favoring the use of water in the treatment of patients.

Dr. Sears also spoke in favor of the water cure in case of fever.

Dr. Hill, of Austin, spoke briefly on the paper, saying that he used the water cure in many cases successfully, but at other times it was far from successful, and he was of the opinion that circumstances should govern their use.

Dr. Q. C. Smith, of Austin, spoke on the subject advocating intelligence and familiarity in the use of the remedy in question before its appliance.

Dr. Menafee spoke in reference to the use of the coal-tar cures, also saying they should not be condemned without a full knowledge of their powers, for when a full knowledge is gained it becomes evident that they are of some use in certain cases. He was of the opinion, however, that the water cure was at times ineffective.

Dr. Wallace closed the argument stating that possibly in trying to be brief he had failed to make himself plain. That he endorsed most all that had been said and admitted that circumstances and knowledge must go with the use of the cure.

### JUDICIAL COUNCIL.

Credentials from the following Societies were reported received, and delegates recommended to be accredited as follows:

E. L. Menafee, Hood County Medical and Surgical Association; Z. T. Bunday, N. B. Kennedy, M. D. Knox, and J. Bine, Hill County Medical Society; A. B. Gardner, Austin County Medical Society; W. E. Fowler and J. H. Powell, from Bastrop Medical Society.

The report of the council was adoped. Mr. S. L. Newton presented his certificate as a delegate to the present Convention from the Texas State Pharmacentical Association.

On motion, his certificate was accepted, and he was given the privileges of the Convention hall.

Adjourned till 9 a. m. Wednesday.

---

### SECOND DAY—MORNING SESSION.

---

### SECTION ON MEDICAL JURISPRUDENCE.

The Section on Medical Jurisprudence being called by President Sears, Chairman B. F. Brittain, of Arlington, read his report as chairman of that Section. On motion the paper was referred to the Publishing Committee without debate.

The Section was opened with a paper on

### MIND CURE, by Dr. D. R. Wallace, of Waco. At this point a recess of a few minutes was taken for the selection of a Nominating Committee, so they might get to work.

[The Nominating Committee is composed of one representative from each affiliating society and one from each county where there is no medical society; names omitted.—Ed.]

The next paper was that of Dr. E. D. Capps, of Fort Worth, touching on the subject of

MORPHINE SUICIDES—PREVENTION—TREATMENT OF OPIUM POISONING.

On motion, the paper was referred to the Publishing Committee.

Dr. Kennedy, of Galveston, spoke briefly on the merits of the paper, as did also Dr. Cerna, of Galveston; Dr. Wooten, of Austin; Dr. Warfield, of Galveston, and Dr. Osborne, of Cleburne.

THE "AMICKS."

Dr. Denton, at this point, offered a resolution as follows:

WHEREAS, A secret nostrum manufacturing concern known as the Amick Chemical Company, located at Cincinnati, O., have for the past several months flooded the United States with circulars of printed matter directed to almost every physician throughout the country, and to many thousands of private persons, claiming to cure tubercular consumption and some other diseases by the use of a secret compound known as the Amick Chemical Cure; and

WHEREAS, This concern has by fraud made illegitimate use of the Associated Press dispatches, and have used the names of men eminent in the profession, without their consent, for the purpose of advertising their said secret nostrum and for the purpose of deceiving the people throughout the country; and

WHEREAS, The claim has been made and circulated broadcast, that this secret nostrum, has the endorsement of the members of this Association, and of the regular profession generally, as well as the reputable medical journals throughout the country; and

WHEREAS, These sharpers, engaged in the manufacture of this secret nostrum, with consummate skill and shrewd money-making judgment, have undertaken, with unblushing effrontery, to use the medical profession in the distribution and sale of their nostrums by refusing to send their so-called chemical cure to patients except through the medical attendant; therefore be it

*Resolved*, By the Texas State Medical Association, that we denounce the statement that the so-called chemical cure has the endorsement of this Association, or any reputable member of it, as false; and the peculiar and reprehensive methods of advertising adopted by this concern, and the attempt to use the members of the profession in the distribution and sale of a secret nostrum, is hereby condemned and denounced, and that we advise the members of this Association, and the profession generally throughout the country, to refuse their aid to this concern in the distribution and sale of their secret nostrum, which should be treated, in all respects, as other secret and patent medicines.

After some discussion the resolution was adopted and ordered printed in all the medical journals in the country.

County Medical Societies were called on for report of progress, and delegates from the following societies made very satisfactory reports:

Johnson Co. Medical Society.....................— members
Hill Co. Medical Society .......................45 members
Galveston Co. Medical Society...................30 members
W. B. M. (Williamson, Burnet and Bell.)..........— members
Collin Co. Medical Society......................40 members
All in flourishing condition.

A circular was read by Dr. West, the Secretary, referring to the meeting of the American Medical Association in San Francisco, June 4th. The trustees have selected the Santa Fe route through Colorado. All Texas physicians who expect to attend this meeting, in order to secure accommodation on the Association train, should advise W. A. Tuley, general passenger agent, Dallas, Texas, not later than May 20. A special sleeper will leave Galveston at 6:30 a. m.; Fort Worth, at 8:15 p. m., running through to San Francisco without change, and will be attached to the Association train at Newton, Kansas, May 29.

---

### AFTERNOON SESSION.

Section on Surgery recalled; Dr. T. J. Bell, Chairman.

Dr. M. M. Smith read a paper on "Brain Surgery of To-day." Referred.

Dr. Suttle, of Corsicana, read a paper on "Contusion of the Brain," and Dr. Hibbs, of New York, also read a paper. [The reporter regrets that he was not able to be present on this occasion, and failed to get a minute of the proceedings of the early part of the session.]

Dr. J. Sam'l Price, of Beaumont, reported six cases of compound fracture of the frontal bone.

Dr. Hadra, of San Antonio, arose to a discussion of the three papers, advancing some of his personal experiences thereon, and making a hurried review of the theories advanced by his contemporaries.

Dr. Scott, of Temple, arose to compliment Dr. Price on his paper, but also stated that it was quite frequently the case that remedies applied to skull fractures were not at all times sufficient or adequate to the importance of the fracture, and that he was of the opinion that the remedies applied were not heroic enough in many cases.

Dr. Mathews, of Austin, spoke on Dr. Smith's paper, reviewing the same, briefly criticising on parallel lines.

Dr. Thompson, of Galveston, also spoke in reference to the various papers, giving his personal experience in reference to similar cases.

Dr. Cummings, of Austin, spoke on the subject, advocating a full review of the subject, so that all might have the benefit of it.

Dr. J. H. Reuss, of Cuero, presented some of his cases on this subject, and the effects of an operation performed thereon by himself.

Quite a number of other gentlemen spoke on this subject, giving their experiences thereon.

The Section on Dermatology was not called, there being but one paper on the program, that by Prof. Isadore Dyer, M. D., of Tulane University, N. O., on

THE USE AND ABUSE OF ARSENIC IN SKIN DISEASES, but instead, on motion, the regular order of business was suspended, and Dr. Dyer was invited to read his paper at this point.

Dr. Alexander, of Coleman, arose to congratulate the author on his paper, but thought it advisable to warn all patients that the use of arsenic at first produces bad effects, but constant use will bring all things right.

The paper of Dr. Dyer was accepted with thanks, and referred to the Publishing Committee.

At this point the work of the afternoon was taken back to the Section on Obstetrics and Diseases of Children, and with Dr. Gardner, the Secretary, in the chair temporarily. Dr. J. F. Y. Paine, of Galveston, read his paper on "The Present Status of Symphysiotomy and its Relation to Other Obstetrical Operations." On motion, the paper was received, and referred to the Publishing Committee.

Dr. M. M. Smith, of Austin, being next on the program, presented a report of a monstrosity without limbs or sexual organs. On motion, the paper was received, and referred to the Publishing Committee.

Dr. Edwards, of the University, and Dr. Lacy, of San Antonio, both spoke in explanation of the monstrosity mentioned in Dr. Smith's paper, advancing various reasons for its cause.

Dr. Hill, of Austin, stated that he had had a similar case under his own care not long since.

Passing on to the

SECTION ON OPHTHALMOLOGY AND OTOLOGY,

Dr. R. H. Chilton, of Dallas, presented a paper on "Muscular Insufficiency—Errors of Refraction." The paper was referred to the Publishing Committee.

The paper of Mr. H. L. Hilgartner, of Austin, on blindnes in Texas, was next in order, and engaged deep interest.

Drs. Carhart and Warfield spoke at some length on Dr. Hilgartner's paper, after which it was referred to the Publishing Committee.

The next paper under consideration was that of Dr. B. F. Church, of Terrell, on the subject of "Indications and Plea for the Mastoid Operation." On motion, the paper was received, and referred to the Publishing Committee.

Dr. Dulaney, of Tennessee, spoke on the subject briefly, imparting some valuable information from personal experience.

Dr. Tyner, of Austin, spoke briefly on the paper also.

Dr. Church closed the debate, briefly answering some objections and queries brought up against his paper.

Dr. T. J. Tyner, of Austin, made a brief reference to a paper read by him at the International Congress of Doctors, at Rome, Italy, and on motion it was ordered that the paper in question be referred to the Publishing Committee.

Drs. R. Gentbruck, of Houston, and J. D. Jordan, of Madisonville, tendered their resignations as members of the Association. The resignations were accepted, and the Convention stood adjourned at 6 o'clock, until 8 o'clock. The President's address, and annual oration by Dr. Blailock, were delivered Wednesday night at 8 o'clock.

---

### THIRD DAY—MORNING SESSION.

---

Section on State Medicine and Public Hygiene was called. Dr. T. J. Bennett, Chairman, read his address on

### THE NATION'S SIN OF OMISSION.

In this paper the writer showed that every citizen, however humble, who yields obedience to the laws, supports the government, pays his taxes and performs all duties required of him as a citizen has a right to protection by the government, not only in his life, liberty and property, but against dangers that threaten his life; and amongst the greatest of dangers he mentioned certain diseases known to be *preventable;* and pointing out in startling statistics the immense numbers of lives lost every year by neglect of hygiene—the absence of laws to enforce sanitation—argued that the government is responsible for this unnecessary loss of life, and that the people have a right to demand protection from preventable sickness—from quack doctors—from mad dogs and from all other similar and dissimilar dangers. He made a strong plea for sanitary legislation, and showed how thousand of lives can be saved by it; instancing reforms instituted in Chicago by legislative enactment based upon report of Boards of Health; and, as the American Medical Association is the only representative National body, its petition to Congress to create a Department of Public Health should be taken as the medical *vox populi* [or the "vox" of the medical "*populi*"]. Until a head of the medical profession is created, the doctor argued, no progress can be made in National sanitary legislation.

The paper was referred to the Pnblishing Committee, but on motion of Dr. Ashton, of Dallas, the Secretary was instructed to send a copy—under the seal of this Society, to each senator and member of Congress from Texas. Thereupon Dr. Carhart introduced the following

RESOLUTION.

Recognizing the necessity for additional and advised legislation by the National government for the protection of the public health against diseases other than cholera, small-pox and yellow fever, and which diseases science has demonstrated to be preventable by proper attention to sanitation, the Texas State Medical Association hereby endorse the action of the American Medical Association in its petition to Congress to create a department of health with an executive officer at its head with equal power and dignity with that of the executives of the other departments of the government.

We believe that the creation of such a department is absolutely necessary as a first step in the great reform in internal sanitation, the necessity for which is made manifest by statistics, which show everywhere the fearful loss of life through diseases which can be prevented, and we claim that the citizen who pays his taxes and renders obedience to the laws of the country, has a right to the protection of his health as well as of his person, property and other rights, natural or acqnired.

*Resolved*, That the neglect to make laws for the protection of the public health against preventable diseases, in accordance with the revelations of sanitary science, is attributable to the want of definite information on the part of Congress as to the requirements, and not to indifference or disregard of the rights of the public to such protection; and that in the absence ot a head of the public interest, the action of the American Medical Association should be taken as an expression of medical opinion on this subject.

The above resolution was adopted, after a brief discussion, by a unanimous vote, and at the suggestion of Dr. Bennett, who was in the chair, a motion was made and carried, ordering that copies of the resolution be sent the Texas representatives in Congress.

Section on Practice recalled:

Dr. Carhart read his paper on Typhoid Fever; referred.

Dr. Clarence Warfield, of Galveston, read a paper on

### CLINICAL NOTES ON TYPHUS FEVER IN MEXICO.

[The reading of Dr. Warfield's paper was interrupted by expiration of time and was concluded next day; referred to Publishing Committee after an animated discussion. The paper was very creditable to the young gentleman.—Ed.]

---

### NIGHT SESSION.

The afternoon of the third day was spent in an excursion on Lake McDonald, the great inland lake created by Austin's famous granite dam. Some three hundred doctors and their ladies, besides a large number of ladies of the city, went up on the great side-wheel steamer Ben Hur. It was a delightful trip, and as there was a fine brass band on board, lively music contributed to the enjoyment and many young persons tripped on the light fantastic.

Returning to the city a night session was held for installation of officers, and memorial service.

The President delivered his address and Dr. Blailock, the orator of the occasion, his oration, on Wednesday night. The Journal regrets not being able to publish these addresses in full.

The following are the officers elect, reported by the Nominating Committee Thursday morning:

President, J. W. McLaughlin; First Vice-President, W. L. York; Second Vice-President, W. R. Blailock: Third Vice-President, W. H. Lancaster.

Judicial Council—Drs. J. H. O'Hara, Link, Loggins, J. M. Inge.

Orator—C. M. Rosser.

Section on Practice of Medicine—Chairman, J. W.Carhart; Secretary, J. H. Frey.

Section on Obstetrics and Diseases of Children—Chairman, A. B. Gardner; Secretary, A. H. Schenk.

Section on Surgery—Chairman, A. C. Walker; Secretary, E. W. Capps.

Section on Medical Jurisprudence—Chairman, F. S. White; Secretary, B. F. Church.

Section on State Medicine, Etc.—Chairman, R. Rutherford; Secretary, I. N. Suttle.

Section on Gynecology—Chairman, J. E. Gilchrist: Secretary, E. L. Menafee.

Section on Dermatology—Chairman, S. E. Hudson; Secretary, M. M. Smith.

Section on Ophthalmology and Otology—Chairman, R. E. Moss; Secretary, W. M. Yater.

Section on Microscopy and Pathology—Chairman, A. J. Smith; Secretary, J. M. Frazer.

The Publishing Committee was left standing.

Section on Necrology—Chairman, Dr. Oliver; Secretary, E. C. Nettles.

Commissioner of Board of Health—R. M. Swearingen.

The next place of meeting, Dallas, the 4th Tuesday in April, 1895.

Committee of Arrangements—Chairman, J. O. McReynolds.

The President and Vice-Presidents elect were inducted into office Thursday night, each returning thanks for the honor, etc., except Dr. W. L. York, First Vice-President, who was absent. Dr. Osborn spoke for him. The new President took the chair and announced that the hour had been appointed for memorial services.

Tributes of respect were paid to the memory of the members who, since last meeting, crossed over the Stygian river, and appropriate resolutions were adopted, which resolutions will duly appear in the volume of Transactions. The deceased members are Drs. C. F. Paine, of Comanche; H. W. Waters, of Independence; Jno. L. Wagley, of Cleburne; C. L. Mays, of Stevensville, and H. M. Oliver, of Ennis. Dr. Coleman's tribute to Dr. Paine, and Dr. Osborn's remarks touching the life and character and the loss of the lamented Dr. Wagley were eloquent and touching indeed.

After memorial service an attempt was made to pass a resolution on bidding for practice, but it was ruled out of order.

### NEW MEMBERS, 1894.

The Judicial Council reported favorably on the following, and they were admitted to membership:

| NAME. | POSTOFFICE AND COUNTY. |
|---|---|
| Abney, G. M | Franklin, Robertson |
| Autrey, J. L | Luna, Freestone |
| Blackburn, J. H | Mineral Wells, Palo Pinto |
| Boatner, J. W | Lewisville, Denton |
| Boyd, Frank D | San Antonio, Bexar |
| Boyer, S. S | Throckmorton, Throckmorton |
| Brown, J. E | McGregor, McLennan |
| Burleson, J. H | Austin, Texas |
| Caston, Wm | Corsicana, Navarro |
| Fowler, W. E | Bastrop, Bastrop |
| Frey, J. H | Corsicana, Navarro |
| Fuller, F. A | Jacksonville, Cherokee |
| Gammon, Wm | Galveston, Galveston |
| Granbury, H. B | Austin, Travis |
| Hill, H. B | Austin, Travis |
| Hill, L. D | Webberville, Travis |
| Hons, J. M | San Marcos, Hays |
| Hubbard, M | Raceland, Collin |
| Jones, E. A | Osage, Coryell |
| Knox, T. R | Hallettsville, Lavaca |
| Ledbetter, A. A | Hallettsville, Lavaca |
| Lutrell, J. M | Mineral Wells, Palo Pinto |
| Miller, J. E | Lockhart, Caldwell |
| Morris, J. E | Madisonville, Madison |
| Morris, Seth M | Galveston, Galveston |
| McCaleb, G. W | Gonzales, Gonzales |
| McReynolds, John O | Dallas, Dallas |
| Scott, A. C | Temple, Bell |
| Shropshire, W | Houston, Harris |
| Smith, B. D | St. Elmo, Travis |
| Strayhorn, J. M | Bartlett, Williamson |
| Warfield, C | Galveston Galveston |
| Wickline, R. M | Johnson City, Blanco |
| Williams, J. O | Wm. Penn, Washington |

### AN IMPORTANT REPORT.

The following report was made by the Judicial Council Wednesday p. m.:

DR. SEARS, PRESIDENT—In the matter of controversy between the West Texas Medical Society and Dr. James Kennedy, the Judicial Council beg to report, after a careful consideration of the subject and all the facts brought to the attention of the Council, we respectfully submit that the action of the Council, at the annual meeting of the State Medical Association at Galveston, April, 1893, approving the expulsion of Dr. Kennedy by the West Texas Medical Society, and requesting that said expulsion be reconsidered on conditions stipulated, to-wit, "that Dr. Kennedy apologize for language used," and that "the West Texas Medical Society withdraw an offensive expression applied to Dr. Kennedy by said West Texas Medical Society," was altogether advisory and not intended to rescind the expulsion of Dr. Kennedy, nor to claim for this Association the right of dictating qualifications of membership to affiliating societies or subordinate associations. While we regret that the request for adjustment of the difficulty was not complied with on the part of the West Texas Medical Society, we can not do more than has already been done in the interest of harmony. The former action of the West Texas Medical Society, in the expulsion of Dr. Kennedy, is approved. Dr. Kennedy having complied with all the requirements imposed by the Judicial Council of 1893, we respectfully ask that he be restored to membership in the State Medical Association.

Respectfully submitted,

M. D. KNOX, Chairman.

The report raised quite a discussion pro and con, the Chair and some of the members holding that the restoration of Dr. Kennedy to the membership of the State Medical Association expelled all the members of the West Texas Medical Society, inasmuch as an endorsement of him was a condemnation of the West Texas Medical Society. It was explained that Dr. Kennedy was now a member of the Galveston Medical Society, and his reinstatement would have nothing at all to do with the West Texas Medical Society. With the understanding that its adoption would not in any way reflect on the West Texas Medical Society, the report was adopted with few dissenting votes.

### FOURTH DAY—MORNING SESSION.

President McLaughlin in the chair.

Dr. S. R. Burroughs offered the following resolution which was adopted:

That the Texas State Medical Association does not endorse the majority report of the Committee on Revision of the Code of Medical Ethics appointed by the American Medical Association, and that the delegates to the latter be and are hereby instructed to oppose any change in the present Code.

SECTION ON GYNECOLOGY.

On motion, the Section on Gynecology was taken up, and Dr. Geo. W. Christian, as chairman of that section, read his report. The paper was, on motion, referred to the Publishing Committee, without debate.

Dr. J. F. Y. Paine, of Galveston, was next under this section, with a paper on "Intra-Uterine Manipulations in Pelvic Inflammation." In lieu of the reading of the same, Dr. Paine made an oral explanation of his paper, in a very clear and concise manner, that was attended with marked pleasure and interest on the part of the delegates. On motion, the paper of Dr. Paine was referred to the Publishing Committee.

Dr. McDaniel spoke briefly on the merits of the paper, giving his experience.

Dr. Cunningham also referred briefly to the merits of the subject.

Dr. Gordon and Dr. Blailock endorsed Dr. Paine's paper most heartily.

Dr. Christian, as chairman of the section, considered Dr. Paine's paper of that merit that deserved a few remarks of endorsement from himself, which were cheerfully given.

Dr. M. D. Knox also endorsed the paper.

Dr. West, of Galveston, stated that the plan suggested by Dr. Paine was in direct contradiction to cerain well known doctors' treatment, but as far as he was concerned, he heartily endorsed Dr. Paine's paper.

Dr. Paine closed the discussion, as author of the paper.

Dr. J. Cummings, of Austin, then presented a paper on "An Original Method of Disposing of the Ligatures after Laparotomies in the Pelvic Region, Effecting perfect Drainage, with report of cases."

On motion, the paper was referred to the Publishing Committee.

Dr. Paine spoke briefly on the paper.

Dr. W. R. Blailock presented a paper on "Hysterectomy with Unexpected Complications." The paper was referred to the Publishing Committee.

This section was closed by referring the balance of the papers to the Publishing Committee by caption. On motion, the

SECTION ON MICROSCOPY AND PATHOLOGY

was taken up. In the absence of the chairman, Dr. A. J. Smith, Dr. David Cerna acted as chairman.

A paper was received from Dr. Herff, of San Antonio, and was referred to the Publishing Committee without reading.

The report of Chairman Dr. A. J. Smith was also referred without reading.

Professor Charles L. Edwards was next on the program, by invitation, and delivered a very exhaustive lecture on Experimental Biology. The paper was, on motion, referred to the Publishing Committee, with the thanks of the Convention to its author.

Dr. William Gammon, of Galveston, was next on the program, with a paper on "Some Clinical Observations on Hæmatozoa Malariæ. On motion, the paper was referred to the Publishing Committee. The paper was discussed by several delegates.

Dr. J. H. Frey, of Corsicana, delivered a paper on "Gunshot Wounds." The paper was referred to the Publishing Committee.

A resolution was passed thanking the Arrangement Committee of the local fraternity for the efficient and highly satisfactory manner in which they had entertained the visitors.

In reference to the resolution offered by Dr. Osborn at the meeting Thursday night, Dr. E. L. Menefee, of Granberry, Texas, offered the following resolution:

*Resolved*, That we, the State Medical Association, consider as unethical, and therefore disapprove and condemn the custom in some sections of the State of physicians bidding against each other for the medical practice of any community, organization, county jail, or poor farm, or for private families.

The resolution was adopted.

A resolution was next offered by Dr. Cerna, of Galveston, as follows:

In the year 1892 Dr. Solomon Solis-Cohen offered to the Philadelphia County Medical Society a set of resolutions condemning the apparently growing disobedience of the code of ethics of the American Medical Association by members of the regular profession. The resolutions were upheld by said Philadelphia Medical Society. They were soon afterwards presented and unanimously adopted, also, by the Medical Society of the State of Pennsylvania. Slightly modified to meet our own requirements, I offer these resolutions for the adoption of which I ask the support of each and every member of our organization.

*Resolved*, That the Texas State Medical Association hereby expresses its highest disapprobation of the practice of giving certificates or testimonials to secret preparations alleged to be of medicinal virtue, and calls the attention of the affiliated county societies to the fact that such action on the part of the members of the said societies is in derogation of the ability of the profession, and in violation of the letter and spirit of the code of ethics of the American Medical Association and of this Society.

*Resolved*, That this Association likewise expresses its disapprobation of the practice of inserting advertisements of secret preparations in the columns of medical journals, such action being an insult to the intelligence of the profession, and a degradation of journals indulging therein to the level of the patent medicine almanac. Especially to be condemned is the action of the *American Medical Association Journal* in admitting such advertisements.

*Resolved*, That copies of these resolutions, duly attested by the permanent Secretary, be sent to all the county societies in affiliation with this Association, to the American Medical Association, to State Medical Societies in affiliation therein, and to the publishers and editors of American medical journals.

The resolution passed with two dissenting votes, viz: Drs. J. W. Carhart, of Lampasas, and Clarence Warfield, of Galveston. These two gentlemen voted against the resolution, giving as their reason for so doing that they had no reliable information that the *Americal Medical Association Journal* published any such advertisements as was charged. These gentlemen agreed to the body of the resolution with this exception.

Dr. Daniel asked the privilege of a personal remark with reference to publication of advertisements of certain preparations. He said, since the medical journals had been impeached, he felt as if he should say a word in defense of the TEXAS MEDICAL JOURNAL. The JOURNAL was on record, for many years, as a staunch and persistent advocate of the code, and a strict compliance with its letter, and that it would be very unbecoming in the management, to admit to its columns even a questionable advertisement; but the great difficulty was to draw the line and say where the permissible stops and the forbidden begins; and still more difficult to say who shall draw the line; who be the censor of the medical press advertising business. The leading journals of the country set the fashion, and we smaller ones feel licensed and safe in taking such as we see in the *Journal of the American Medical Association* and the *New York Medical Record*, and other great publications. The management had tried conscientiously to keep within the line of legitimate business, and had persistently refused all others; that the JOURNAL was the first journal in America to denounce the Amick Chemical Cure when it first appeared in the *Lancet-Clinic*—even before the editor of that journal had explained how it got in there; and yet that concern had the audacity only yesterday to offer us its advertisement. We indignantly refused it, of course.

Dr. E. L. Menefee, and several other gentlemen, spoke kind words for the TEXAS MEDICAL JOURNAL, and said it deserved the assistance and support and encouragement of the Texas profession for the great work it has done. For which remarks we beg to tender our sincere acknowledgments.

An invitation was received and accepted from the Mississippi Valley Medical Association at Hot Springs, inviting the Convention to visit that body at its next annual meeting.

Dr. Carhart stated that the Faculty of St. Mary's Academy would be at home to the Convention at 3 o'clock.

Dr. Blailock tried to get through a resolution doing away with the annual banquet at each Convention, but the majority killed it.

All the papers on the program not read before the convention, were ordered referred to the Publishing Committee by caption.

The Convention then on motion adjourned to meet the fourth Tuesday in April of next year at Dallas.

NOTES OF THE CONVENTION.

Dr. Hibbs, of New York City, a Texas born and bred young doctor, and a handsome, dignified gentleman, honored the Association with his presence and read a most excellent paper.

Prof. W. H. Bell, of the Jefferson Medical College, Philadelphia, honored the Association with his presence also, and read a paper on the "Embarrassments of Medicine," which was listened to with deep interest. Dr. Bell is a nephew of the late Judge Bell, of Austin.

